# Diffuse Maculopapular Dermatitis Associated With Leuprorelin Acetate Androgen Deprivation Therapy

**DOI:** 10.7759/cureus.65207

**Published:** 2024-07-23

**Authors:** Kwabena Boahen Asare, Nirav S Kapadia

**Affiliations:** 1 Department of Dermatology, Dartmouth Cancer Center, Dartmouth Health, Lebanon, USA; 2 Department of Radiation Oncology, Dartmouth Cancer Center, Dartmouth Health, Lebanon, USA

**Keywords:** prednisone, prostate cancer, leuprorelin acetate (leuprolide), androgen deprivation therapy (adt), diffuse maculopapular dermatitis

## Abstract

Androgen deprivation therapy (ADT) is one of the effective treatment methods for prostate cancer, often used with radiation therapy. Among the key ADT agents is leuprolide, a synthetic gonadotropin-releasing hormone agonist, which effectively suppresses testosterone production which is a requisite for the growth and division of prostate cancer cells. However, leuprolide is associated with several well-known side effects and less common dermatological reactions. In this case, we present an 80-year-old male patient with stage IIB prostate cancer who developed diffuse maculopapular dermatitis following leuprolide acetate ADT. The patient first experienced mild dermatitis following the fifth monthly 7.5 mg leuprolide injection before it developed into a general body rash after six injections. The dermatitis manifested on the patient’s arms, thighs, calves, dorsum, and back of hands but sparing the abdomen, face, and neck. The pruritic dermatitis was managed successfully with a three-week course of prednisone which led to complete resolution without long-term sequelae. This case highlights the importance of recognizing and managing dermatological side effects associated with ADT. Clinicians should maintain an index of suspicion and act promptly when these side effects manifest. Systematic reporting and further research are essential to enhance patient safety and understanding of drug-related dermatological manifestations.

## Introduction

Androgen deprivation therapy (ADT) is a key treatment method for the treatment of patients with advanced or metastatic prostate cancer and may be used with other treatment methods such as radiotherapy [1-3]. Among the effective ADT agents is leuprolide, also known as leuprorelin acetate, a synthetic agonist analog of gonadotropin-releasing hormone (GnRH). Leuprolide functions by suppressing the production of testosterone, which is crucial for prostate cancer cell growth [4]. By reducing testosterone production, leuprolide stops or slows down the advancement of prostate cancer, particularly in cases where the cancer is hormone-sensitive [5,6]. Common side effects of leuprolide ADT include fatigue, hot flashes, sexual side effects, susceptibility to metabolic disorders, muscle loss, weakness, osteoporosis, and coronary heart disease [7-9]. While cases of dermatological reactions associated with leuprolide have been reported, papular eruptions remain widely underreported [9-20]. Here, we present an unusual case of a patient exhibiting diffuse maculopapular dermatitis associated with leuprorelin acetate ADT.

## Case presentation

**Figure 1:**
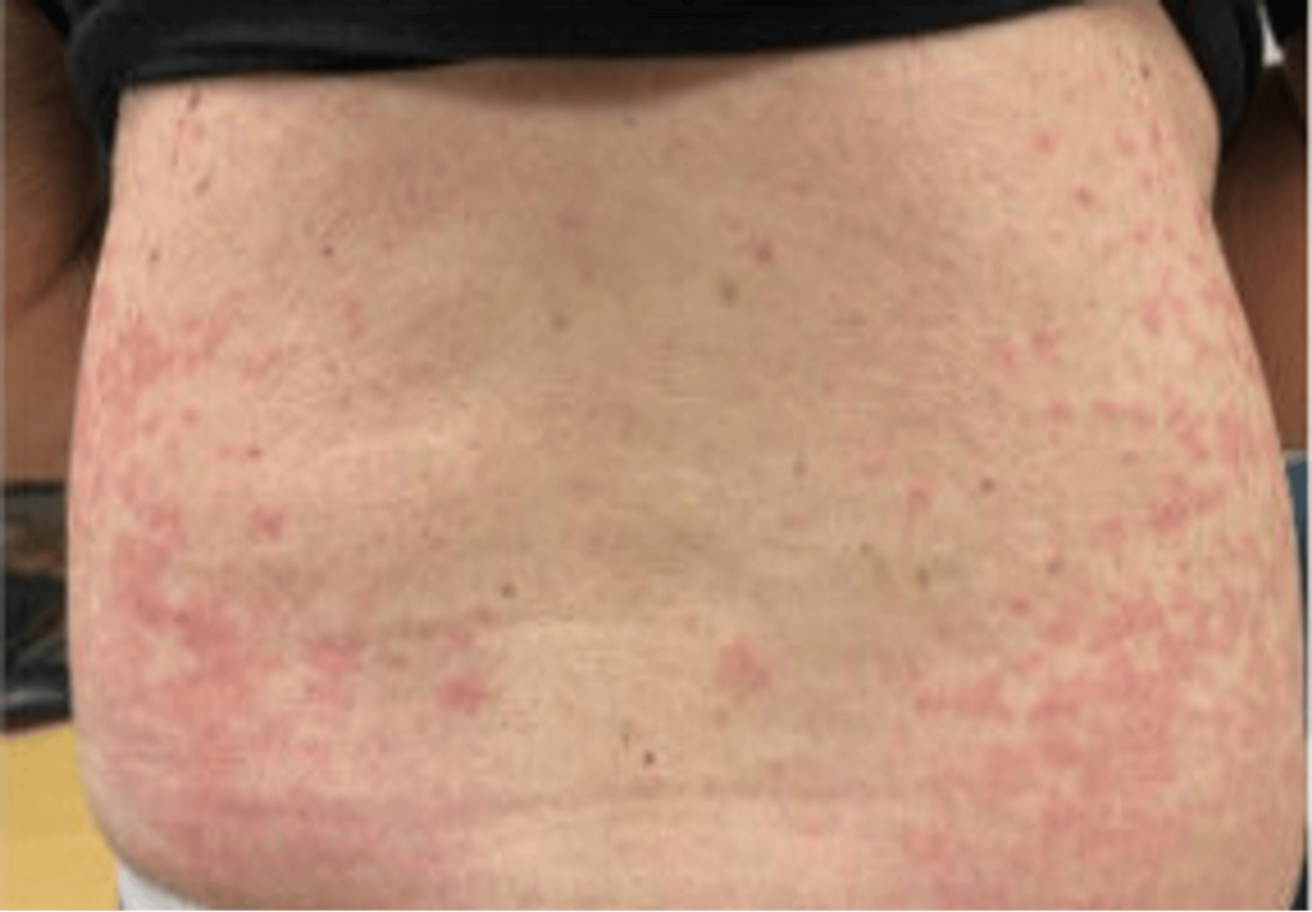
Photograph of maculopapular dermatitis after the fifth intramuscular injection of 7.5 mg dose of leuprolide. The image shows dermatitis on the patient’s dorsum.

**Figure 2:**
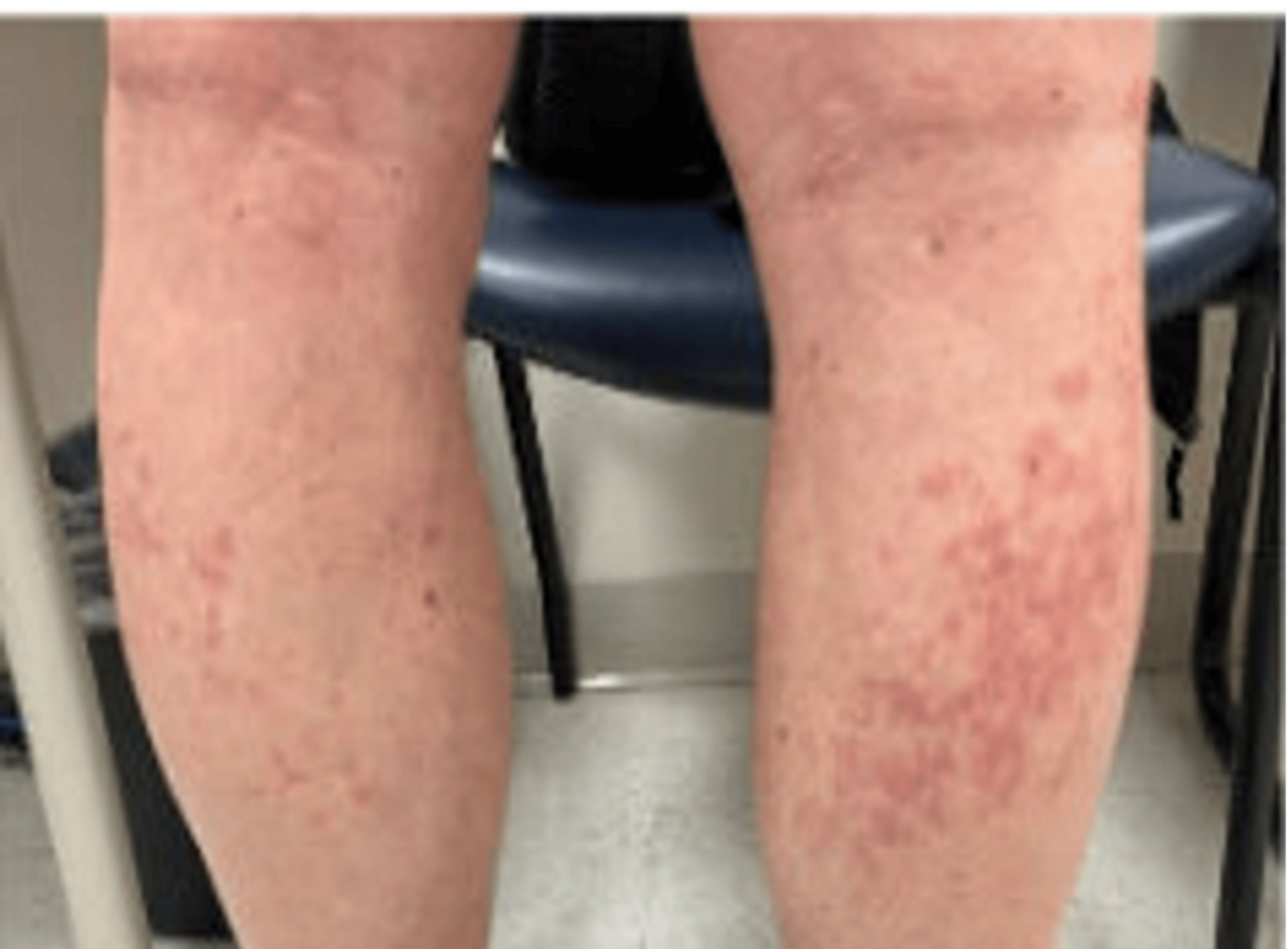
Photograph of maculopapular dermatitis after the fifth intramuscular injection of 7.5 mg dose of leuprolide. The image shows dermatitis on the patient’s calves.

**Figure 3:**
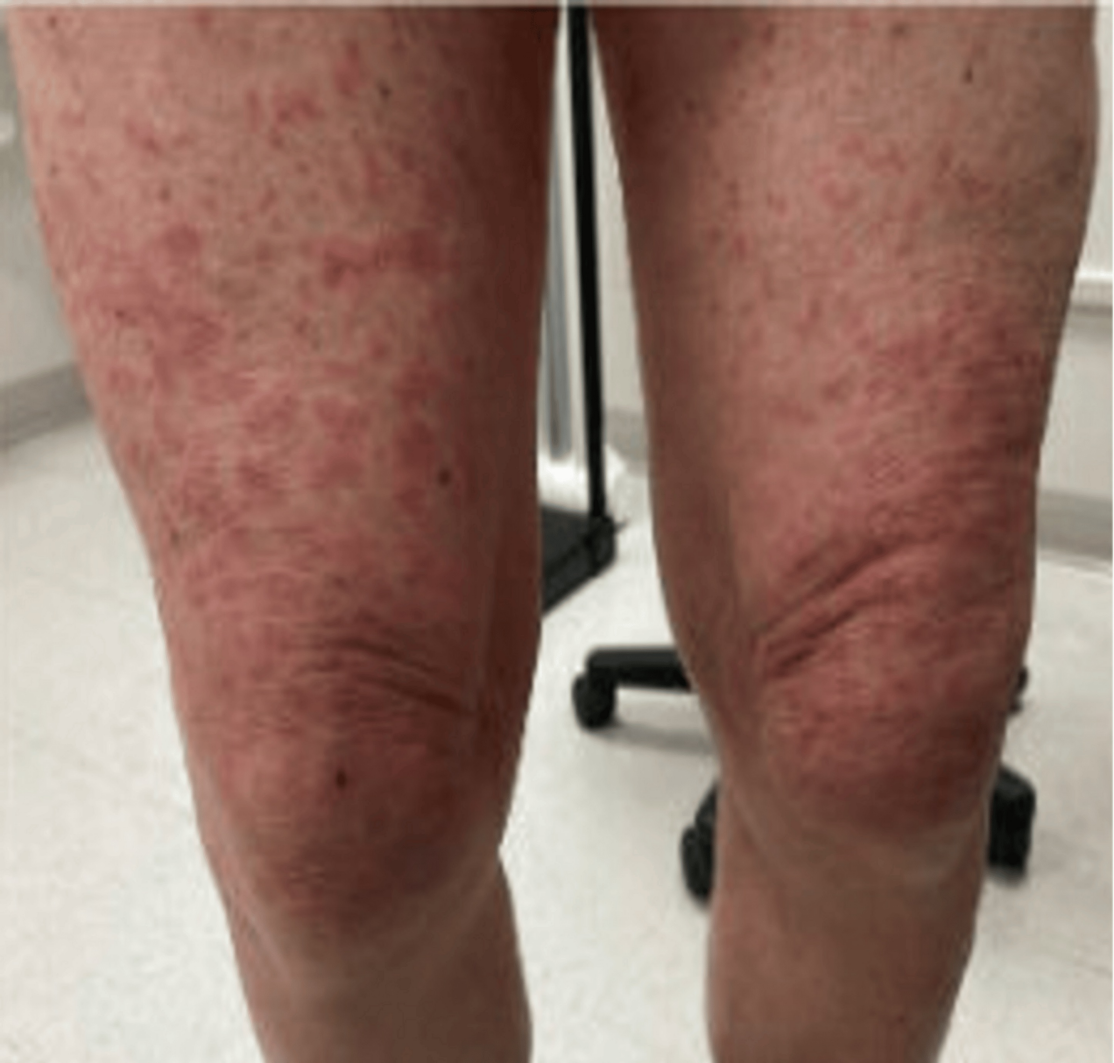
Photograph of maculopapular dermatitis after the fifth intramuscular injection of 7.5 mg dose of leuprolide. The image shows dermatitis on the patient’s thighs.

**Figure 4:**
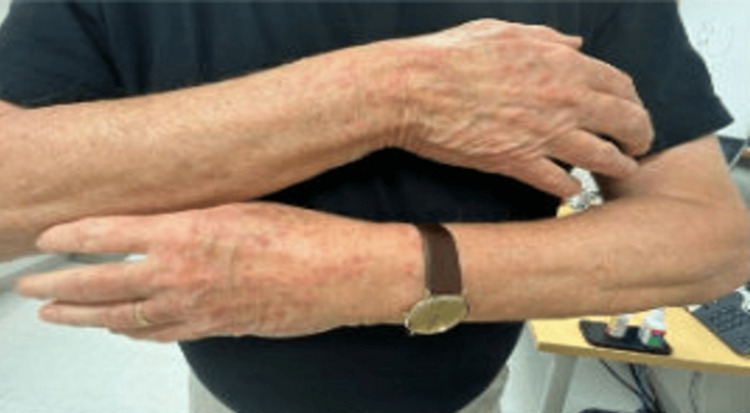
Photograph of maculopapular dermatitis after the fifth intramuscular injection of 7.5 mg dose of leuprolide. The image shows dermatitis at the back of the patient’s hands.

An 80-year-old patient diagnosed with stage IIB, unfavorable, intermediate-risk prostate cancer underwent leuprorelin acetate ADT, receiving 7.5 mg doses of leuprorelin every month. Following the fifth injection of leuprorelin acetate, the patient developed mild dermatitis on the arms. Given its mild nature, no further management was recommended. Two days following the next injection, however, the rash developed into a diffuse maculopapular dermatitis on the patient’s arms, thighs, calves, dorsum, and back of his hands (Figures 1-4). However, his abdomen, face, and neck were spared.

Given the pruritic nature of the dermatitis, the patient was given prednisone 20 mg, twice daily for three weeks, followed by a rapid taper. Over the course of the steroid treatment, dermatitis and pruritus improved and eventually healed with no long-term visible sequalae. No further injections of leuprorelin acetate were planned or administered.

## Discussion

Prostate cancer is one of the most commonly diagnosed tumors and ranks as the sixth leading cause of cancer-related deaths among men [1]. Standard treatment methods for prostate cancer include surgery and radiation therapy, the latter of which is often combined with ADT either in the short (e.g., 4-6 months) or long (12-36 months) term [1,2]. The choice of treatment depends on various factors, including cancer stage, the patient’s overall health and preferences, and potential side effects [3]. In this case, the patient received planned short-term neoadjuvant and concurrent ADT, alongside radiotherapy to his pelvis and prostate.

While the efficacy of leuprorelin acetate in suppressing testosterone production and slowing the progression of prostate cancer is well-established [4-8], its potential dermatological side effects, particularly diffuse maculopapular dermatitis, are less recognized and underreported in clinical practice [9-20].

The onset of dermatitis in our patient following the fifth leuprorelin acetate injection highlights the essence of monitoring patients undergoing ADT for potential adverse reactions. Although the initial rash was mild, its progression to diffuse maculopapular dermatitis necessitated intervention to alleviate symptoms and prevent further complications. Systemic steroidal treatment with prednisone proved effective in managing diffuse dermatitis, leading to significant improvement and eventual resolution of the skin reactions.

This case contributes to the growing body of evidence documenting dermatological manifestations associated with leuprorelin acetate ADT. While previous reports have documented papular eruptions linked to leuprorelin acetate, our case highlights a rare development of diffuse, symptomatic maculopapular dermatitis, expanding the spectrum of dermatological adverse effects attributed to this medication.

The underlying mechanisms behind the development of dermatitis in response to leuprorelin acetate remain unclear and warrant further investigation. It is plausible that immune-mediated processes or hypersensitivity reactions may play a role in the pathogenesis of dermatological side effects associated with this medication or, more likely, one of its components. A previous report, for instance, established that papuloerythroderma, a skin condition related to maculopapular dermatitis, is linked to eosinophil invasion, and mediated by T-helper 2 cells [20].

## Conclusions

This case highlights the importance of recognizing and managing dermatological adverse effects associated with drug treatments. Clinicians should remain vigilant for such reactions and be prepared to intervene promptly to optimize patient outcomes. Additionally, systematic reporting of adverse drug reactions and further research are crucial for enhancing patient safety and improving our understanding of drug-related dermatological manifestations.
